# Postrelease monitoring habitat selection by reintroduced burchell's zebra and blue wildebeest in southern Mozambique

**DOI:** 10.1002/ece3.5221

**Published:** 2019-05-03

**Authors:** Luís Comissário Mandlate, Ezidio da Lucia Cuamba, Flávio H. G. Rodrigues

**Affiliations:** ^1^ Department of General Biology, Postgraduate Programme in Ecology, Conservation and Wildlife Management, Instituto de Ciências Biológicas Universidade Federal de Minas Gerais – UFMG Belo Horizonte Brazil; ^2^ Agricultural Division, Forestry Engineering Course Instituto Superior Politécnico de Gaza Chókwè Mozambique; ^3^ Department of Botanic, Faculty of Natural Sciences Universidade Lúrio Pemba Mozambique

**Keywords:** habitat, reintroduced, wildebeest, zebra

## Abstract

**Aim:**

In Africa, reintroduction of wild animal species to conservation areas is a common practice, for the recovery or restoration of populations. Effective monitoring of reintroduced species requires understanding of basic ecological requirements such as habitat selection of these species in the new environment. Therefore, the present study investigated the habitat selection of zebra and wildebeest following their reintroduction into Maputo Special Reserve, south Mozambique, and we use binary logistic analyses to investigate the relative influence of biotic and abiotic factors in determining the habitat use.

**Location:**

Maputo Special Reserve, south Mozambique.

**Methods:**

We conducted the study from July 2016 to June 2017. The data were collected by direct observation, driving the vehicle along the reserve's roads that covered the vegetation communities where zebras and wildebeest are known to commonly occur. Habitat selection was calculated using selection indices (Manly's alpha), and binary logistic analyses were used to investigate the relative influence of biotic and abiotic factors in determining the habitat use.

**Results:**

The arboreal savanna was the preferred habitat by both herbivore species. Habitat use of zebra appeared to be strongly determined by characteristics such as high grass cover, high grass greenness, and distance to water, while the habitat use by wildebeest, was strongly affected by grass height.

**Main conclusions:**

Both zebra and wildebeest prefer arboreal savanna, forage selection likely drove preference of this habitat. Greater grass cover and greater percentage greenness of the grass both significantly increased the odds of zebra use of habitat, whereas the odds of use decreased with increases in distance to water, meaning an opportunity to ingest large amounts of grass biomass with higher quality, and this opportunity decreases with increasing in distance to water. Grass height was in the highest‐ranking model predicting habitat use by wildebeest, and during the dry season the use of habitat increased with increasing grass height, suggesting that selecting areas with tall grasses by wildebeest equated to choosing areas with higher grass quantity, as the food intake rate increases with grass height.

## 1 INTRODUCTION

The study of habitat selection is one of the most crucial topics to understand the biological requirements, conservation, and management of animals **(**Groom & Harris, 2009). It is also a prerequisite to understand the distribution and abundance of animals to determine the possible species for reintroduction, as well as the density, which can be stocked (Dekker, Rooyen, & Bothma, 1996).

Effective monitoring of reintroduced species requires understanding of basic ecological requirements such as habitat selection (Muposhi et al., 2014). Habitat quality of the release site has an impact on survival of species and their ability to adapt to the new environment (Rantanen, Buner, Riordan, Sotherton, & Macdonald, 2010). In the context of the ecosystem, a clear understanding of the factors influencing habitat selection is considered a prerequisite for interpretation of interactions between animals and their environment (Gandiwa, 2013; O'Kane, 2005).

Many landscape‐scale models of herbivore distributions focus primarily on the role of the biotic factors, such as forage quality and quantity (Redfern, Rant, & Iggs, 2003). However, a number of other biotic and abiotic factors including shelter from extreme conditions (Owen‐Smith, 2002; Traill, 2003), refuge from predators (Owen‐Smith, 2002), cover, slope, availability of water, and interactions with other species, in particular, competition (Bailey et al., 1996; Prins et al., 2006), are equally as important and can act as the primary determinants of the selection of habitats by herbivores (Groom & Harris, 2009).

Preferred habitats are those that confer the highest fitness and thus support the highest equilibrium density in the absence of other confounding factors, such as competition and predation (Riginos & Grace, 2008). Habitats with higher vegetation cover and tree density decrease the visibility and ability of herbivores to detect predators and avoid them.

Herbivore species such as zebra and wildebeest tend to avoid closed woodland habitats (Apps, 2000; Owen‐Smith, 2002; Riginos & Grace, 2008), whereas species such as sable, not documented to successfully fight against large predators, should avoid being detected by predators by using habitats providing adequate woody cover for concealment (Macandza, 2009). Otherwise, large trees improve grass quality by enhancing below‐canopy grass nutrients, such as nitrogen and phosphorus contents, therefore attracting and benefiting grazers (Treydte, Heitkönig, & Ludwig, 2009).

Seasonal fluctuations in water availability cause changes in the profitability of habitats and the animals must be able to adapt behavioral and spatial responses to these fluctuations (Bennitt, Bonyongo, & Harris, 2014).

In Africa, the reintroduction of wild animal species, to conservation areas, is a common practice, for the restoration of populations (Rantanen et al., 2010). The Maputo Special Reserve (MSR) is one of the conservation areas in Mozambique from which the populations of same wild herbivores were nearly extirpated, between 1977 and 1992, as a result of the civil war (Stalmans, 2015). A multiyear reintroduction program was initiated in 2010 to restore the reserve's populations of zebra *Equus quagga* and wildebeest *Connochaetes taurinus* using stock obtained from parks in South Africa and Swaziland (Hanekom & Cumbane, 2016). These species play a fundamental role in the ecosystem, its conservation, and its potential as a tourist destination (Stalmans, 2015).

Redfern et al. (2003) argue that a combination of both biotic and abiotic factors may be particularly important in determining the habitat selection of large herbivore in African savanna ecosystems. The present study is the first to analyze habitat selection of zebra and wildebeest following their reintroduction into Maputo Special Reserve, southern Mozambique. It was expected that zebra and wildebeest would select different habitat type in proportion to their availability in the reserve.

We also use binary logistic regression analyses to investigate the relative influence of biotic and abiotic factors in determining the habitat use of these two herbivore species. The predictions made included: (a) zebra and wildebeest are obligate drinkers (Apps, 2000).Therefore, we predicted that: for both herbivore species, the odds of use habitat will increase with decreasing distance to water, (b) zebra have narrow muzzle well suited for clipping tall grass, whereas the wildebeest have wide muzzle well suited for clipping short grass (Arsenault & Owen‐Smith, 2008; Estes, 1991). Thus, we predicted that: the odds of use habitat by zebra will increase with increasing the grass height and decreases with increase the grass height for wildebeest, (c) green grass leaves have higher concentration of protein, minerals, and soluble carbohydrates than brown leaves (Groom & Harris, 2009; Owen‐Smith, 1982). Therefore, we predicted that: the odds of use habitat by zebra and wildebeest will increase with increasing the greenness of the grasses, (d) in African savannas, the density and distribution of grazers is primarily determined by grass cover (Gandiwa, 2013). However, the grass cover increases where tree cover is low (Riginos & Grace, 2008). Therefore, we predicted that: the odds of use habitat by zebra and wildebeest will increase with increasing grass cover and decreasing with increases the tree cover, (e) in over much of African savanna, the topography is a series of undulations, in which the rain falling on the undulations runs off slopes into the depressions, so, the grass in the depressions tends to be taller and more green (Bell, 1971). Therefore, we predicted that: the odds of use habitat by zebra and wildebeest will increase with decreasing the elevation.

## 2 METHODS

### 2.1 Study area

**Figure 1:**
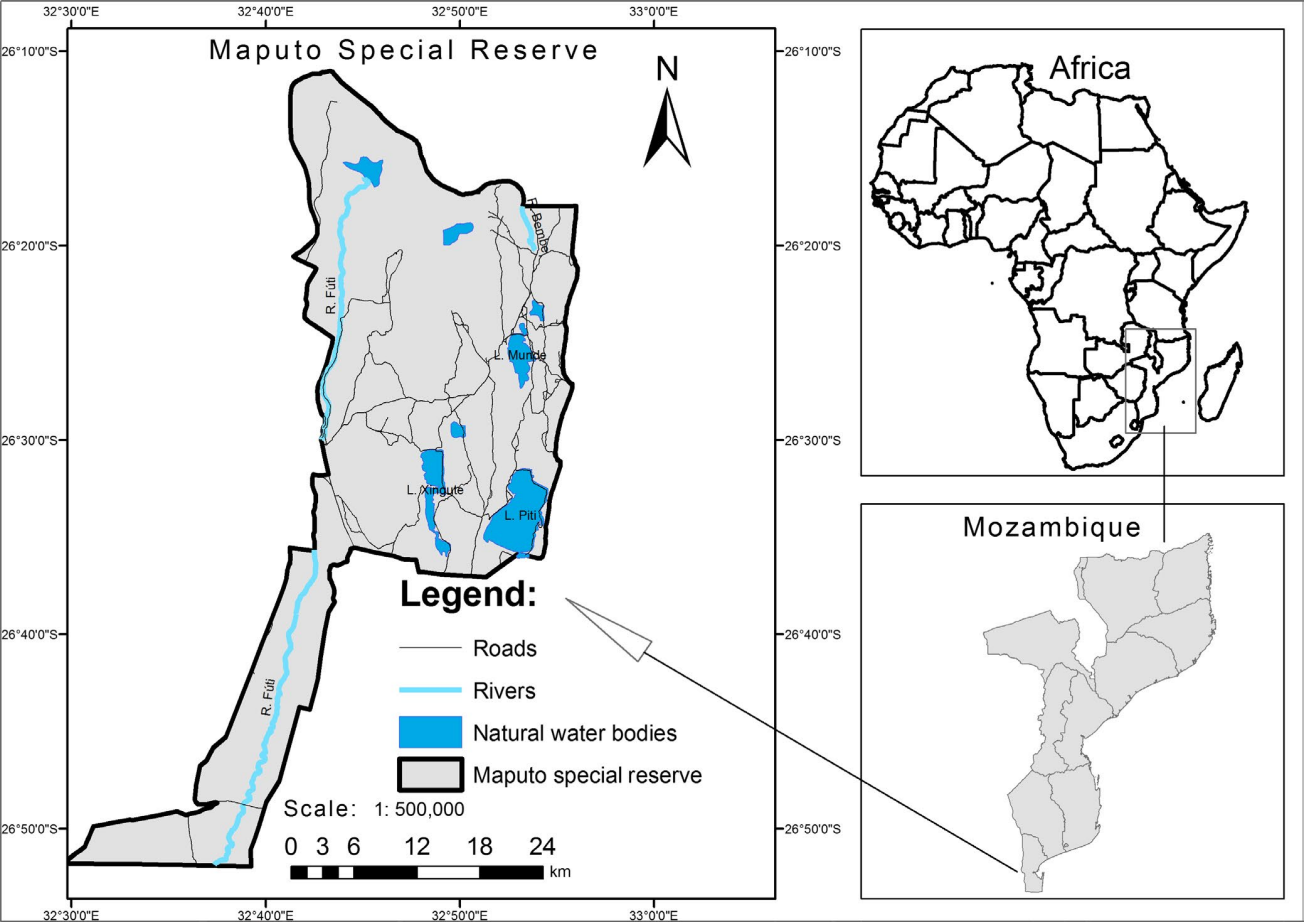
Location of Maputo Special Reserve, southern Mozambique

The study was carried out at the Maputo Special Reserve (MSR), in southern Mozambique (26°25′S, 32°45′E). The reserve was established in 1932 and has an area of 1,000 km^2^ (Figure 1). The local climate has two distinct seasons; a hot rainy season (October‐March) and a cooler dry season (April‐September). The annual rainfall varies from 690 to 1,000 mm (De Boer, Ntumi, Correia, & Mafuca, 2000).

According to Marzoli (2007), there are nine principal vegetation communities in MSR (Appendix S1), including (a) eucalyptus plantation, (b) coastal dense wood vegetation, (c) semideciduous open forest, (d) semideciduous forest, (e) semi‐evergreen forest, (f) shrub savanna, (g) arboreal savanna, (h) riverine vegetation, and (i) mangroves that border the bay and surround the deltas of the Maputo river and Bembe canal (De Boer et al., 2000).

Since the reintroduction program initiated in 2010, blue wildebeest and zebra have increased significantly from 276 to 351 and from 303 to 446, respectively (Hanekom & Cumbane, 2016). Other herbivore species in the Reserve include about 400 African elephant (*Loxodonta Africana*), 2,611 common reedbuck (*Redunca arundinum*), 405 red duiker (*Cephalophus natalensis*), 257 gray duiker (*Sylvicapra grimmia*), 255 bushbuck (*Tragelaphus scriptus*), 200 impala (*Aepyceros melampus*), 350 kudu (*Tragelaphus strepsiceros*), 100 warthog (*Phacochoerus aethiopicus*), 255 bushbuck (*Tragelaphus scriptus*), and 230 nyala (*Tragelaphus angasii*) (Hanekom & Cumbane, 2016).

### 2.2 Research design and data collection

We applied study design I (Thomas & Taylor, 1990), whereby we assessed the resource selection at the population level, as the individual animals were not identified. The data were collected between July 2016 and June of 2017 and were grouped into two seasons: dry season (July–September 2016), wet season (October 2016–March 2017), and dry season (April–June 2017).

The roads of the reserve that cover different vegetation communities (Appendix S1) were surveyed using a vehicle, following standard routes. These standard routes were devised and formalized after a ground reconnaissance session that aimed to determine the areas on the Reserve where zebra and wildebeest were most likely to occur, and the areas that were inaccessible to them. This resulted in some areas of the reserve being excluded from analysis as the terrain in these areas was found to be inaccessible to both herbivore species.

During 10 days each month, the zebra and wildebeest were monitored from the vehicle, driven at a speed of 25 km/h, carrying two observers, one of whom monitored the presence of herds along the right side of the road, and the other who monitored the left side of the road, both using binoculars (Zenith TEMPEST 8 × 30, 7.5o field). This monitoring was conducted during peak ungulate feeding times, in the morning (6 h30−10 h00) and afternoon (15 h30−18 h30). A total distance of 2,353 km was surveyed, with a mean distance of 196 km/mo. We sampled as many herds as possible, but did not return to the same areas on a given day, to minimize the possibility of resampling a herd on the same day.

When a herd was encountered, the vehicle was stopped. A laser range finder (RZ900D Laser Distance Meter 6X) was used to determine distance from the observer to the herd. Once the animals left the site, the research team immediately approached the site on foot. The position of the site was recorded using GPS (GPS map XL, Garmin 62).

At each sampling site, a quadrat 0.7 × 0.7 m was placed, and two additional quadrats were placed at each of the four cardinal points (with a total of eight), at intervals of 2 m from the first quadrat. In these, plots were searched for freshly grazed grass to examine if the herds were grazing or not. This was done as we assumed that the habitat used by zebra and wildebeest were influenced primarily by the distribution of forage than any other factor. Freshly grazed grass was identified by the lighter, more vivid coloration of the broken leaves and stems in comparison with older grazing (Macandza, Owen‐Smith, & Cain, 2012). For subsequent analysis, grazing/ not grazing of grasses at each sampling site was generated by coding 1 for grazing and 0 for not grazing.

The scale of analysis is important for the reliable assessment of habitat selection (Novellie, 1990). In the present study, habitat selection was analyzed at macroscale level, where the coordinates of all sampling sites were overlaid on a map of the vegetation of the MSR to identify which habitat type was selected by animals. This map was produced in ArcGIS 9.3, based on the data of forest inventory of Marzoli (2007). In addition, at a microscale level (Novellie, 1990), we measured seven microhabitat variables in the field that could potentially influence the distribution of herbivore species (Bailey et al., 1996).

The variables were measured within the 25 m radius plots and included: (a) altitude—measured as the height above sea level in meters at the site, determined from a topocadastral map with the GPS recording used for verification, (b) slop—steepest slope in degrees recorded using GPS, (c) woody cover—visually estimated as the proportion of the site shaded or covered by tree (height ≥ 2.5 m) and grouped into the following classes: 0; 1–10; 11–25; 26–50; and 51–75, (d) grass height (cm)—measured from the ground to the tip of the highest leaf of the predominant grass species, (e) grass greenness—visually estimated as the proportion of green leaves available in the sward within the plot and classified according to the eight‐point scale (Walker, 1976): 0%, 1%–10%, 11%–25%, 26%–50%, 51%–75%, 76%–90%, 91%–90%, and 100%, (f) grass cover—visually estimated as the proportion of area within the 25 m radius plot covered by grass and classified according to the eight‐point (Walker, 1976), and (g) in addition, the distance from the center of the 25 m radius plot to the nearest water point was calculated using the range finder and calculated using Arc GIS 9.3 for points that were impossible the measured using the range finder.

The minimum distance between sampling points was 200 m. A total of 244 points were sampled for zebra and 120 for wildebeest. The number of sampling points per road depended on the location of the animals. The data were collected in five habitat types in which the herbivore herds were encountered: eucalypt plantation (2,781.95 ha), arboreal savanna (127,081 ha), shrub savanna (131,862 ha), semi‐evergreen forest (16,765.49 ha), and semideciduous open forest (661,490.88 ha).

### 2.3 Data analysis

#### 2.3.1 Habitat selection

For each season, habitat selection by each herbivore was estimated by applying the method described by Neu, Byers, and Peek (1974), using a chi‐square goodness‐of‐fit analysis to determine whether there was a significant difference between expected habitat use (the proportions of the different habitats available in the reserve) and the observed frequency of usage of each habitat type. Bonferroni confidence intervals (*p* < 0.05 significance) were used to determine which habitat types were being preferred or avoided significantly (Krebs, 2014). The confidence intervals were calculated by:

$$Pi\pm Z_{1-\alpha /2k}{\left[Pi(1-Pi)/n\right]}^{1/2}$$

where *Pi* = the observed proportion of use of habitat type *i*, *k* = the total number of habitat types, *Z*_1−_ *_α_*_/2_ *_k_* = the *Z* score based on the α level (0.05) for *k* habitat types, and *n* = the total number of observations of the herbivore species during the respective season.

A habitat was considered to be preferred if the lower limit of the confidence interval exceeded the proportion available for that habitat in the study area. If the available proportion of the habitat fell within the confidence interval, a habitat was considered to be used in proportion to its availability, that is, no preference. If the available proportion of the habitat exceeded the upper level of the confidence interval, the habitat was considered to have been avoided by the herbivore (Krebs, 2014).

#### 2.3.2 Model for predicting habitat use

For each sampling site, to obtain the single value of wood cover estimated following Walker (1976), we allocated the midpoints value of that class recorded. The same approach was done for the grass greenness and grass cover. The data recorded and coded as 1 for grazing and 0 for not grazing, was used as the dependent variable in the logistic regressions, with seven independent variables included in the models (see Table 1), to estimate the probability of use of a site. We considered 16 a priori candidate models (see Table 2), to establish whether use by zebra and wildebeest was associated with particular variables (Table 1). A priori models were developed to evaluate which variable played an important role on habitat use both independently and in combination. All independent variables were included in the models, because no colinearity among them was found (Zar, 2009).

**Table 1:** Summary statistics (means ± *SE*) of the variables included in the logistic regressions models

Variables	Codes of variables	Burchell's zebra		Blue wildebeest	
		Dry season	Wet season	Dry season	Wet season
Woody cover (%)	WC	7.01 ± 1.49	6.30 ± 1.19	8.97 ± 2.27	7.91 ± 2.23
Grass greenness (%)	GG	48.23 ± 2.93	45.31 ± 3.15	60.69 ± 4.39	81.00 ± 3.27
Grass height (cm)	GH	72.46 ± 4.88	73.43 ± 3.74	81.09 ± 6.13	62.22 ± 5.07
Grass cover (%)	GC	61.57 ± 2.93	51.64 ± 2.51	58.76 ± 4.40	66.1 ± 3.80
Distance to water (m)	DW	720 ± 60.90	512 ± 40.32	1591.67 ± 358.47	584.33 ± 82.62
Slop (^o^)	SL	89.89 ± 0.008	89.88 ± 0.006	89.86 ± 0.02	89.87 ± 0.09
Elevation (m)	EL	23.34 ± 0.62	23.08 ± 0.56	20.84 ± 0.97	20.81 ± 0.91

**Table 2:** Priori candidate model for predicting habitat use by reintroduced burchell's zebra and blue wildebeest in Maputo Special Reserve, Mozambique

Models
Grass cover
Grass height
Grass greenness
Wood cover
Distance to water
Slop
Elevation
Grass cover + Grass height
Grass cover + Grass greenness
Grass height + Grass greenness
Grass cover + Grass Height + Grass greenness
Grass Height + Grass cover + Distance to water
Grass Height + Grass greenness + Distance to water
Grass cover + Grass greenness + Distance to water
Grass Height + Elevation+Distance to water
Slop + Grass greenness

The Akaike Information Criterion (AIC) was used to select the most‐parsimonious models (Burnham, Anderson, & Huyvaert, 2011). The best models were selected using ΔAICc, and model with ΔAICc ≤ 2 AICC, were considered to have equivalent support (Burnham & Anderson, 2004). Model uncertainty was accounted for by calculating model‐averaged parameter estimates (±*SE*) using multimodel averaging across all a priori models (Symonds & Moussalli, 2010). Odds ratios and 95% confidence intervals were derived by exponentiation of the model‐averaged parameter estimates (O'Shaughnessy, Cain, & Owen‐Smith, 2014; Symonds & Moussalli, 2010).

## 3 RESULTS

### 3.1 Habitat selection

The observed frequency of use of different habitat types differed significantly from that expected in both dry and wet season, for both zebra and wildebeest (in the dry season, for zebra: *χ* ^2^ = 549.5, *df* = 4, *p* < 0.0001; wildebeest: *χ* ^2^ = 33.2, *df* = 1, *p* < 0.0001; in the wet season, for zebra: *χ* ^2^ = 525.9, *df* = 4, *p* < 0.0001; wildebeest: *χ* ^2^ = 299.8, *df* = 4, *p* < 0.0001). The Bonferroni test confirmed that zebra and wildebeest preferred arboreal savanna in all periods of the study (Table 3). In the dry season, 85% of the zebra observation were recorded in arboreal savanna, increasing to 90% in the wet season. For wildebeest, an even higher observation rate (89%) was recorded in arboreal savanna in the dry season, although this declined to 79% in the wet season (Table 4).

**Table 3:** Habitat selection by zebra and wildebeest in Maputo Special Reserve using Bonferroni simultaneous confidence intervals

Season	Habitat type	Expected proportion of usage (Oi)	Observed proportion of usage (Pi)	Bonferroni intervals for (Pi)	Preference
Burchell's zebra					
Dry season	Eucalyptus plantation	0.001	0.027	−0.003 ≤ *p* ≥ 0.05	=
	Semi‐evergreen forest	0.009	0.018	−0.006 ≤ *p* ≥ 0.042	=
	Shrub savana	0.073	0.090	0.036 ≤ *p* ≥ 0.143	=
	Arboreal savanna	0.070	0.855	0.790 ≤ *p* ≥ 0.921*	+
	Semideciduous open forest	0.367	0.009	−0.008 ≤ *p* ≥ 0.026*	−
Wet season	Eucalyptus plantation	0.001	0.022	−0.002 ≤ *p* ≥ 0.048	=
	Semi‐evergreen forest	0.009	0.022	−0.002 ≤ *p* ≥ 0.048	=
	Shrub savanna	0.073	0.053	0.014 ≤ *p* ≥ 0.091	=
	Arboreal savanna	0.070	0.893	0.840 ≤ *p* ≥ 0.946*	+
	Semideciduous open forest	0.367	0.007	−0.007 ≤ *p* ≥ 0.022*	−
Blue wildebeest					
Dry season	Shrub savanna	0.073	0.113	0.028 ≤ *p* ≥ 0.199	=
	Arboreal savanna	0.070	0.887	0.801 ≤ *p* ≥ 0.972*	+
Wet season	Forest plantation	0.001	0.045	−0.004 ≤ *p* ≥ 0.094	=
	Semi‐evergreen forest	0.009	0.045	−0.004 ≤ *p* ≥ 0.094	=
	Shrub savanna	0.073	0.090	0.021 ≤ *p* ≥ 0.157	=
	Arboreal savanna	0.070	0.791	0.693 ≤ *p* ≥ 0.888*	+
	Semideciduous open forest	0.367	0.030	−0.010 ≤ *p* ≥ 0.070*	−

* Significant selection value: no preference (0), avoidance (−), preference (+).

**Table 4:** Five highest ranking a priori models for probability of use of habitat by zebra and wildebeest relative to environmental characteristics in Maputo Special Reserve, Mozambique

Model	*K*	AICc	Δ AICc	Weight	Log likelihood
Zebra					
Dry season					
GC+GG+DW	4	111.78	0.00	0.22	−51.65
GC+GG	3	112.06	0.28	0.19	−52.89
GC	2	113.22	1.44	0.11	−54.54
GC+GH+GG	4	113.56	1.78	0.09	−52.54
GH+GC+DW	4	114.12	2.34	0.07	−52.82
Wet season					
GG	2	141.87	0.00	0.28	−68.88
GH+GG	3	143.02	1.15	0.16	−68.40
GH+GG+DW	4	143.75	1.88	0.11	−67.69
GC+GG	3	143.76	1.89	0.11	−68.77
SL+GG	3	143.93	2.07	0.10	−68.85
Widebeest					
Dry season					
GH	2	59.43	0.00	0.17	−27.56
GH+GG	3	60.19	0.76	0.12	−26.78
EL	2	60.60	1.18	0.09	−28.15
GG	2	60.64	1.21	0.09	−28.17
GC	2	60.89	1.46	0.08	−28.29
Wet season					
GH	2	78.37	0.00	0.14	−37.07
GG	2	78.69	0.33	0.12	−37.23
WC	2	79.01	0.64	0.10	−37.39
GH+GG	3	79.25	0.89	0.09	−36.39
DW	2	79.46	1.09	0.08	−37.61

Note

Maximized log likelihoods, number of parameters (*k*), Akaike's information criterion adjusted for small sample size (AICC), ΔAICc, and Akaike weights are given.

### 3.2 Model for predicting habitat use

During the dry season, wildebeest used areas with higher wood cover, grass greenness, grass height, and closer to water, but had lower level grass cover, slope and elevation compared to sites used by zebra (Table 1). During the wet season, wildebeest used areas that were higher wood cover, grass greenness, grass cover, and closer to water, but these areas had lower grass cover, slop, and elevation compared to sites used by zebra (Table 1).

During the dry season, grass cover, grass greenness, distance to water were the most important variables predicting habitat selection by zebra (Table 4). A greater grass cover and greater percentage greenness of the grass increased significantly the odds of zebra using an area, whereas the odds of use decreased with increased distance to water (Table 5). Grass height was in the highest‐ranking model predicting habitat use by wildebeest during the dry season (Table 4), with the odds of use increasing with increasing grass height (Table 5).

**Table 5:** Model‐averaged logistic regression coefficient estimates (*β*), standard errors (*SE*), odds ratios, and 95% confidence intervals for odds ratios for variables included in the best approximating models for the probability of use of habitat by zebra and wildebeest relative to environmental characteristics in Maputo Special Reserve.

Variable	*β*	*SE*	Odds ratio	95% confidence for limits odds ratio	
				Lower CL	Upper CL
Zebra					
Dry season					
Grass cover	0.019	0.008	1.013	1.003	1.035
Grass greenness	0.016	0.009	1.011	0.999	1.034
Distance to water	−0.001	0.000	0.999	0.999	1.000
Grass height	0.005	0.005	1.001	0.995	1.016
Slope	4.912	2.913	1.366	0.451	41,000.32
Elevation	0.084	0.039	1.005	1.007	1.173
Wood cover	−0.016	0.014	0.999	0.957	1.012
Wet season					
Grass cover	−0.003	0.008	0.999	0.980	1.013
Grass greenness	0.016	0.007	1.013	1.000	1.029
Distance to water	0.001	0.001	1	0.999	1.002
Grass height	−0.005	0.005	0.998	0.984	1.006
Slope	0.598	3.130	1.068	0.004	840.062
Elevation	0.019	0.036	1	0.949	1.093
Wood cover	−0.014	0.014	0.999	0.958	1.014
Wildebeest					
Dry season					
Grass cover	0.005	0.012	1.001	0.981	1.029
Grass greenness	0.012	0.011	1.004	0.990	1.034
Distance to water	0.000	0.000	1	0.999	1.000
Grass height	0.011	0.008	1.005	0.996	1.027
Slope	0.225	2.677	1.019	0.007	238.060
Elevation	0.046	0.047	1.005	0.955	1.147
Wood cover	−0.001	0.018	0.999	0.963	1.036
Wet season					
Grass cover	0.005	0.010	1.001	0.986	1.025
Grass greenness	0.012	0.011	1.004	0.990	1.035
Distance to water	0.000	0.000	1	0.999	1.001
Grass height	−0.010	0.008	0.995	0.974	1.005
Slope	0.759	0.010	1.079	0.002	2,199.815
Elevation	−0.004	0.041	0.999	0.920	1.079
Wood cover	−0.018	0.019	0.998	0.947	1.019

Abbreviation: CL: confidence limit.

During the wet season, grass greenness was in the most‐supported model predicting habitat use by zebra (Table 4). The odds of use habitat by zebra was highest in slope, grass greenness, elevation, and distance to water; wildebeest were more likely use areas with higher slope, grass greenness, and grass cover. (Table 5).

## 4 DISCUSSION

Our findings showed that both zebra and wildebeest prefer arboreal savanna, a habitat characterized by a continuous grass sward interspersed by numerous trees. Research indicates that both species prefer open areas with reduced woody cover due to the greater visibility of predators (Apps, 2000). However, the principal predator of these two herbivores, the lion, is absent from the MSR, which may mean that predation would not be the factor determining the preference of these herbivores for open habitats at this site. In this case, forage selection likely drove preference of this habitat in both zebra and wildebeest. This would reflect the dietary importance of grass, which provides more than 90% of the zebra diet (Grubb, 1981) and 95% of the wildebeest diet (Duncan, Foose, Gordon, Gakahu, & Lloyd, 1990), with the contribution increasing where tree cover is low (Riginos & Grace, 2008). We found that, the odds of use of habitat by both herbivore species decreased with increases in wood cover (Table 5), and, the grass *Aristida barbicollis*, which contributed most to the diets of both herbivores, and was most preferred are indeed more available in the most preferred habitat (Mandlate, Arsenault, & Rodrigues, 2019).

In Maputo Special Reserve, during the dry season, the habitat use by zebra was strongly affected by grass cover, grass greenness, and distance to water, with the odds of use decreasing with increasing distance to water, and increasing with grass cover and grass greenness, while in the wet season it was strongly affected by grass greenness. These results are consistent with previous findings suggesting that habitat features, particularly the amount of grass cover; grass greenness and proximity of water have the potential to affect the use of habitat by zebra (Gandiwa, 2013; Groom & Harris, 2009; Redfern et al., 2003). As grazers, zebra density and distribution is primarily determined by grass cover (Gandiwa, 2013). This observation conforms to the findings of Gandiwa (2013), who also found grass cover as an environmental variable strongly correlated with zebra density and distribution, in Zimbabwe.

Other researchers have reported that a greater biomass of grass cover and percentage of greenness of the grass increased significantly the odds of grazers presence in Tsavo ecosystem, Kenya (Groom & Harris, 2009). Grass greenness can be considered a predictor of quality of grasses (Groom & Harris, 2009; Owen‐Smith, 1982). Therefore, grazers are expected to focus their activities moving to areas with green grass because this would be nutritionally advantageous (O'Reagain, 1996; Roux, 2010). Thus, zebra on MSR was selecting areas with higher grass cover and grass greenness, and these grass characteristics were important variables in explaining the observed distribution of zebra.

Not surprisingly, the distance to water showed a strong influence in use of habitat by zebra, as this species is an obligate drinker and drinks at least once a day. Therefore, zebra are seldom found more than 8 km from water (Apps, 2000). Earlier studies have shown a significant influence of water on attracting large herbivores, including zebra, hence altering the species distributions (Ogutu et al., 2010; Redfern et al., 2003). It is possible that lower forage availability close to water during the dry season forced the zebra to select areas further away from sources of water. Personal observations in our study area noted that the grasses in areas near to water (low‐lying areas) were evergreen but very short due the overgrazing, while areas further away from water were not all evergreen, but were very tall. There is evidence that a decrease in grazing pressure tends to occur when moving away from a water source (Groom & Harris, 2009).

This result is consistent with the findings of Redfern et al. (2003), who emphasized that during the dry season when the quantity and quality of food is reduced, zebra experiment a trade‐off between surface‐water constraints and quality or quantity requirements of food.

For wildebeest, the habitat use was strongly affected by grass height. During the dry season, the odds of use of habitat increased with increasing grass height. This might appear contrary to much of the literature, which report that, wildebeest as selective ruminants are known to prefer habitats with shorter grasses (Bell, 1971; Sinclair & Griffiths, 1982), since this often indicates better food quality (Voeten & Prins, 1999). However, in areas with shorter grasses, there was a faily low biomass on MRS (Personal observations). So selecting areas with tall grasses by wildebeest equated to choosing areas with higher grass quantity (Conneely, 2011).

In addition, other researchers have reported that grass height is an important factor that influences habitat selection by herbivores (Laca, Ungar, Seligman, & Demment, 1992). The food intake rate increases with grass height because bite size increases as animals apprehend a bigger volume of herbage through increased bite depth and bite area (O'Reagain, 1996). During the dry season, when forage quantity declines, large grazers concentrate foraging on tall grass patches offering high intake rates (Sinclair & Griffiths, 1982), thus justifying the increasing of the odds of use habitat with increases grass height. Furthermore, this result suggests that, during the dry season, forage quantity is more important than quality for large grazers, such as wildebeest, similar to what was reported by other studies (Bell, 1971; Groom & Harris, 2009).

The prediction that the use of habitat by zebra and wildebeest would increase with decreasing the slopes and elevation was not supported here, as we mentioned above, in our study area, the grasses in lowland areas were evergreen but very short due to the overgrazing, while in areas with high elevations were very tall. So, selecting areas with tall grasses by zebra and wildebeest equated to choosing areas with higher grass quantity, justified by the observed increase in the odds the use of habitat by zebra and wildebeest with increasing the elevations and slopes.

This study showed that zebra and wildebeest prefer arboreal savanna, forage availability appear to likely drove preference of this habitat. The present study results suggest that the habitat use by zebra within Maputo Special Reserve is largely affected by the studied environmental factors, such as grass cover, grass greenness, and distance to water, while for wildebeest, the habitat use by this species was strongly affected by grass height.

It is recognized that several factors that can strongly influence large herbivore density and distribution in savannahs, such as grazing lawn, soils, visual obstruction, road densities, human settlements, rainfall, and management regimes, were not included in this present study. Nevertheless, future studies should therefore examine these environmental factors to tease out their relative strengths on zebra and wildebeest distribution in Maputo Special Reserve.

In general, the findings of this study provide valuable baseline information on habitat use by zebra and wildebeest in MSR that would help managers of the reserve to protect suitable habitats, predict future population distributions and seasonal movements and detect changes that might be occurring in this aspect in order to make effective conservation decisions and measures. This study is also important for the conservation of this area and for similar areas where large grazers are being reintroduced.

## CONFLICT OF INTEREST

None declared.

## AUTHOR CONTRIBUTIONS

L.J.C.M. conceptualized the research design, conducted field data collection, interpreted the data, and wrote the manuscript; E.L.C. contributed to interpretation of data and writing the manuscript; F.H.G.R assisted in the research design conceptualization and contributed to writing the manuscript.

## DATA AVAILABILITY STATEMENT

All the data used (geographic coordinates of the distributions of these herbivore species recorded, abiotic and biotic environmental data recorded, all distributions data of density of these herbivore species) that support the results of this study I will make available from the ORCID Connecting Research and Researchers database: https://orcid.org/0000-0003-3731-0961.
