# Supplementary figures and images for: Postrelease monitoring habitat selection by reintroduced burchell's zebra and blue wildebeest in southern Mozambique
Source: Ecol Evol. 2019 May 3;9(11):6458–67. doi: 10.1002/ece3.5221 (PMC6580300; doi:10.1002/ece3.5221)

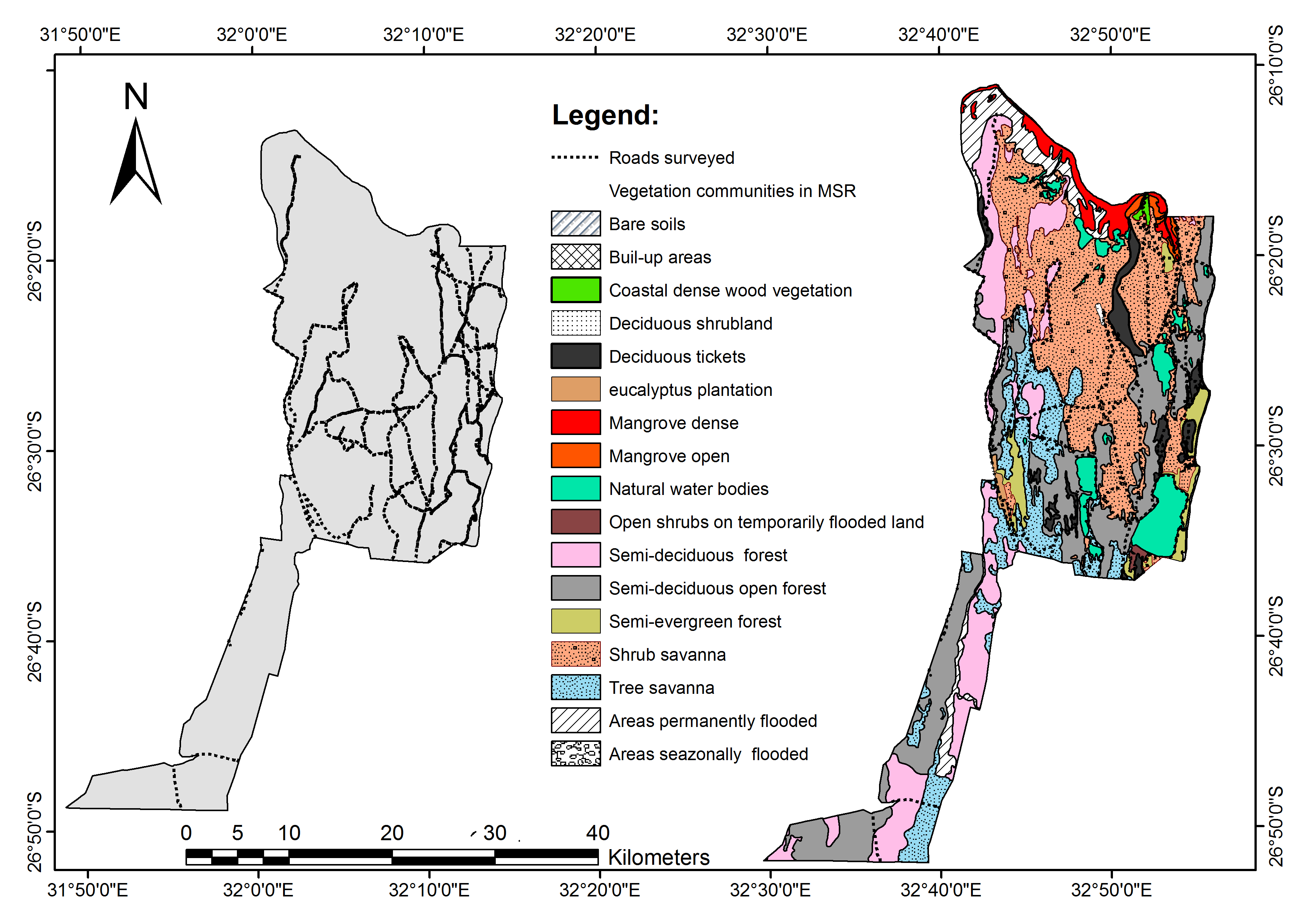

Supplement: Supplementary file 1 [file ECE3-9-6458-s001.tif]
